# Time to diagnosis and determinants of diagnostic delays of people living with a rare disease: results of a Rare Barometer retrospective patient survey

**DOI:** 10.1038/s41431-024-01604-z

**Published:** 2024-05-16

**Authors:** Fatoumata Faye, Claudia Crocione, Roberta Anido de Peña, Simona Bellagambi, Luciana Escati Peñaloza, Amy Hunter, Lene Jensen, Cor Oosterwijk, Eva Schoeters, Daniel de Vicente, Laurence Faivre, Michael Wilbur, Yann Le Cam, Jessie Dubief

**Affiliations:** 1 EURORDIS-Rare Diseases Europe, Paris, France; 2 HHT Europe, Roma, Italy; 3 FADEPOF - Federación Argentina de Enfermedades Poco Frecuentes, Caba, Argentina; 4 UNIAMO – Federazione Italiana Malattie Rare, Roma, Italy; 5 Genetic Alliance UK, London, UK; 6 Sjaeldne Diagnoser - Rare Diseases Denmark, Taastrup, Denmark; 7 VSOP - Vereniging Samenwerkende Ouder En Patiëntenorganisaties, Soest, Netherlands; 8 RaDiOrg - Rare Diseases Belgium asbl/vzw, Brussels, Belgium; 9 FEDER – Federación Española De Enfermedades Raras, Madrid, Spain; 10 Centre Hospitalier Universitaire Dijon-Bourgogne, Dijon, France

**Keywords:** Diagnosis, Social sciences

## Abstract

Timely diagnosis is one of the most serious challenges faced by people living with a rare disease (PLWRD), and this study estimates that in Europe, the average total diagnosis time (TDT) is close to 5 years. We investigated the duration of the TDT for PLWRD in Europe, the difficulties associated with their diagnosis odyssey and the main determinants of diagnosis delays for all rare diseases (RD). We conducted a survey of PLWRD and their families using Rare Barometer, the survey initiative of EURORDIS-Rare Diseases Europe. In geographical Europe, we surveyed 6507 people living with 1675 RD in 41 countries. We then performed a descriptive analysis and ordinal logistic regressions to identify the main determinants of diagnosis delays. Average TDT is 4.7 years. 56% of respondents were diagnosed more than 6 months after a first medical contact. The main determinants of diagnosis delays are symptom onset before 30 years of age, especially during childhood (OR = 3.11; 95% CI: 2.4–4.0) and adolescence (OR = 4.79; 95% CI: 3.7–6.2), being a woman (OR = 1.22; 95% CI:1.1–1.4), living in Northern Europe (OR = 2.15; 95% CI:1.8–2.6) or Western Europe (OR = 1.96; 95% CI:1.6–2.3), the number of healthcare professionals consulted (OR = 5.15; 95% CI:4.1–6.4), misdiagnosis (OR = 2.48; 95% CI:2.1–2.9), referral to a centre of expertise (OR = 1.17; 95% CI:1.0–1.3), unmet needs for psychological support (OR = 1.34; 95% CI:1.2–1.5) and financial support (OR = 1.16; 95% CI:1.0–1.3), having a genetic disease (OR = 1.33; 95% CI:1.1–1.5) and a family history of an RD (OR = 1.36; 95% CI:1.1–1.6). These determinants can inform policies and actions to improve access to diagnosis for all PLWRD.

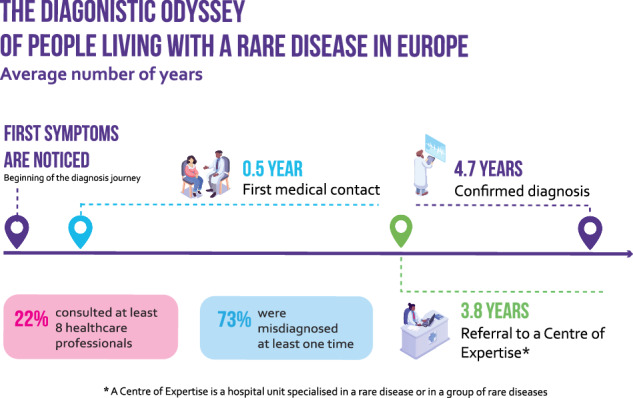

## Introduction

In Europe, rare diseases (RD) have a point prevalence below 5 cases per 10,000 inhabitants. Over 6000 distinct RD have been identified to date, and together they affect approximately 30 million people in Europe and 300 million worldwide [1]. An estimated 72% of RD are genetic and 70% have a paediatric onset [1].

Accurate and timely diagnosis is critical to end the diagnostic odyssey experienced by PLWRD and ensure their access to appropriate health and social care. Early diagnosis allows to provide supportive holistic care as early as possible, can delay or reduce the apparition of symptoms and impairments, and defer the apparition of co-morbidities. Goals set for the diagnosis of RD range from six months (European Rare 2030 foresight study [2]) to one year (International Rare Diseases Research Consortium – IRDiRC [3]) of coming to medical attention. The available evidence [4–12] is primarily condition-specific or country-specific, but shows that the diagnostic search of PLWRD often involves visits with several healthcare professionals, numerous tests, misdiagnoses or inappropriate treatments, including surgeries, which is why it is often referred to as a ‘diagnostic odyssey’. The introduction of next-generation sequencing should improve the diagnosis of people living with genetic RD who have been undiagnosed for years. Still, these technologies are being implemented at different rates in Europe, varying from countries with long-withstanding programmes granting automatic access, to others where they are still being deployed.

The first aim of this study is to estimate the Total Diagnostic Time (TDT) for all PLWRD in Europe *i.e*., the number of years between symptom onset and the confirmed diagnosis, and to describe the difficulties associated with the diagnostic odyssey. The second aim of this study is to identify the main determinants of diagnostic delays (TDT > 1 year) for PLWRD in Europe while distinguishing patient delays (PD), *i.e*., delays between symptom onset and the first medical contact, and health system delays (HSD), *i.e*., delays between the first medical contact and the confirmed diagnosis.

## Methods

### Survey development

This study was conducted within the Rare Barometer programme, the survey initiative of EURORDIS-Rare Diseases Europe [13]. It relies on an online questionnaire designed based on a review of previous studies on the diagnostic delays for PLWRD [4–12], an online discussion forum conducted between September 29 and October 10, 2021 with 61 patient representatives representing 48 RD and 25 countries, and a Topic Expert Committee composed of 24 researchers, patient representatives and experts in health policies. The Council of National Alliances of EURORDIS-Rare Diseases Europe [14], federating patient organisations from a wide range of diseases in 40 countries, was invited to provide input on the topics and indicators to include in the questionnaire, and the final version of the English questionnaire. This version was tested by 9 RD patients and family members, and translated into 26 other languages by professional translators. 15 translations were reviewed by patient representatives in their respective native language.

### Population and recruitment

This survey targeted patients with an RD and their family members (parents and close relatives) over 16 years of age, including former or recovering patients, worldwide. Respondents under 18 years of age were allowed to answer upon agreement of their legal representative. The questionnaire was distributed online from March to June 2022; respondents were contacted by email as part of the EURORDIS Rare Barometer panel [13] (14,525 participants in March 2022) or through patient organisations, social media posts and Facebook ads, ensuring a wide range of experiences were represented. Data were handled per current data protection legislation and curated to remove ineligible respondents and incomplete questionnaires.

Overall, the questionnaire was completed by 13,307 people living in 104 countries with 1931 RD: 3069 responses were received through the Rare Barometer panel, representing a 21% response rate (3069/14,525), and 10,238 responses were received through social media posts, Facebook ads and patient organisations. This paper will focus on the 6507 respondents from Europe who have a confirmed diagnosis for their RD and who entered the dates of perceived symptom onset and confirmed diagnosis. Those respondents live in 41 European countries with 1684 RD (Additional File 1).

### Survey elements

The questionnaire included 49 questions, among which the month and year of each step of the diagnostic journey, the characteristics of the RD (types of symptoms, name) and the diagnostic journey itself (misdiagnosis, support offered, diagnostic tests conducted), as well as sociodemographic questions for the respondent or for the patient when the respondent was the family member of a patient.

### Data analysis

Disease names were all linked to an Orphacode and coded based on Orphanet data [15] on point prevalence, transmission mode, age of onset and type of RD (classification based on organs or systems affected and the genetic nature of the RD is presented in Additional File 2). Countries were grouped according to macroeconomic indicators such as Gross Domestic Product (GDP), health expenditures as part of the GDP, and health expenditures per capita (Additional File 3). All dates were checked for consistency before calculating time-related variables.

Due to the non-normality of TDT, we performed Kruskal Wallis tests to compare median TDT to patients’ sociodemographic and diagnosis-related characteristics. The independent variables with most significant *p*-values were retained and included in ordinal logistic regressions with three dependent variables (Additional files 4 and 5): (i) TDT based on rounded median value (1 year), and on rounded average and third quartile values (5 years); (ii) the time between symptom onset and the first medical contact based on the rounded median and upper quartile values (respectively 0 and 0.25 years), and (iii) the time between first medical contact and confirmed diagnosis considering rounded median value (1 year), and rounded average and third quartile values (5 years). Finally, we measured the association between the selected independent variables and the three dependent variables through multivariate ordinal logistic regression analysis to identify the main predictors of (i) total diagnostic delays, (ii) patient delays (PD) and (iii) health system delays (HSD). Post-estimation analyses were performed to check the models’ specifications.

## Results

### Characteristics of the respondents

**Fig. 1:**
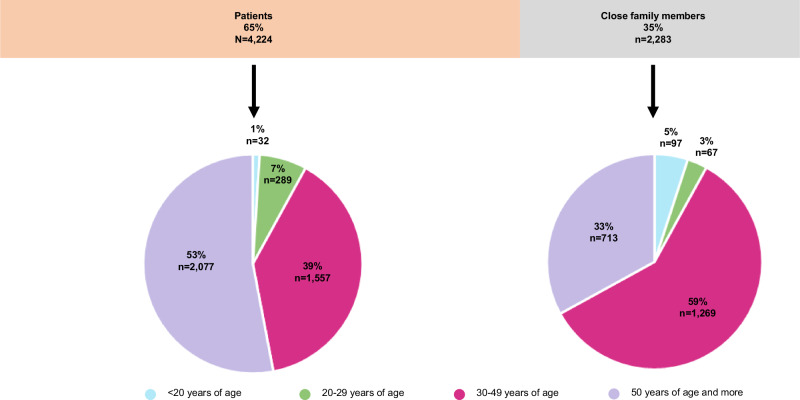
Status of the respondents (patients or close family members) and age at the time of the study for each group. % Percentage, n number of observations. Totals may not be equal between categories because of missing values.

Figure 1 presents some characteristics of the respondents: 65% lived with an RD themselves (4224/6507), and among patients who shared their birth date, 53% (2077/3955) were 50 years of age or more. The remaining 35% (2283/6507) of respondents were close family members of PLWRD, mostly parents (2074/2283) but also spouses (117/2283) or other family members (grandparents, siblings, uncle/aunt or other: 92/2283). 59% (1269/2146) of close family members who shared their birth date were between 30 and 49 years of age.

Of the 49 questions in the survey, 20 independent variables were kept in the ordinal logistic regressions: Table 1 presents their descriptive statistics and TDT. Of note, the point prevalence of the RD (above or below 1/100,000 cases per inhabitant), the respondents’ age at the end of studies and city size did not significantly impact the TDT. The results of the ordinal logistic regressions are presented in Table 2.

**Table 1:** Characteristics of the respondents.

Variable	Category	Distribution	Total diagnosis time in years				
		% (*n*)	Mean	Median (IQR)	% < 1	% 1–4	% ≥ 5
Sociodemographic characteristics							
Age of the patient at perceived symptom onset	<2	25 (1502)	4.9	0.8 (0.1–4.5)	52	24	24
	2–9	11 (666)	8.8	2.0 (0.1–12.0)	42	19	39
	10–19	11 (629)	10.4	5.3 (0.3–18.3)	34	15	51
	20–29	12 (691)	5.5	1.3 (0.2–7.9)	46	22	33
	30–49	28 (1671)	2.7	0.7 (0.1–3.4)	55	26	19
	50 and more	14 (807)	0.6	0.3 (0.0–1.2)	72	22	6
Age of the patient at the time of the study	<2	2 (108)	0.5	0.2 (0.0–0.6)	83	15	2
	2–9	11 (648)	1.2	0.5 (0.1–1.6)	63	32	5
	10–19	12 (709)	2.7	0.9 (0.1–4.6)	50	26	24
	20–29	10 (570)	4.1	1.1 (0.1–6.5)	48	22	30
	30–49	29 (1752)	5.7	1.1 (0.1–7.3)	49	21	30
	50 and more	37 (2206)	6.2	0.8 (0.1–7.0)	51	20	29
Date of the confirmed diagnostic	<2000	12 (775)	2.5	0.3 (0.0–3.3)	61	19	20
	2000–2009	19 (1217)	4.5	0.6 (0.0–4.6)	55	20	25
	2010–2014	18 (1153)	4.8	0.8 (0.1–4.8)	53	22	25
	2015–2019	31 (1989)	5.5	1.0 (0.1–5.8)	49	24	27
	2020–2022	20 (1309)	5.2	1.1 (0.2–5.8)	47	26	27
Gender of the patient	Female	70 (4193)	5.4	1.0 (0.1–6.0)	50	22	28
	Male	30 (1839)	3.7	0.6 (0.1–3.6)	56	23	22
Country Group	Eastern, Central and Southern Europe	16 (989)	3.1	0.5 (0.1–2.7)	59	22	19
	Western Europe	53 (3357)	4.8	0.8 (0.1–5.3)	52	23	26
	Northern Europe	32 (2019)	5.4	1.0 (0.1–6.2)	49	23	28
Characteristics of the diagnosis journey (healthcare system)							
Number of healthcare professionals consulted	0–1	13 (824)	2.5	0.2 (0.0–1.0)	74	13	13
	2–7	65 (4232)	3.5	0.6 (0.1–3.2)	56	24	20
	8+	22 (1451)	9.6	4.6 (0.8–14.8)	26	25	49
Misdiagnosis	No	27 (1751)	2.2	0.2 (0.0–1.2)	73	15	13
	Yes	73 (4756)	5.6	1.3 (0.2–6.9)	44	26	30
Genetic tests	No	39 (2506)	3.9	0.5 (0.1–3.7)	58	25	21
	Yes	55 (3565)	5.6	1.2 (0.2–6.6)	46	25	29
	Don’t know / not relevant	7 (436)	2.9	0.3 (0.0–2.2)	65	20	15
Healthcare professionals reluctant or not sufficiently informed to prescribe genetic tests (declarative)	Yes	23 (1493)	7.7	2.5 (0.3–10.7)	35	24	40
	No	56 (3664)	3.9	0.6 (0.1–3.5)	56	23	21
	Not relevant	21 (1350)	3.8	0.5 (0.1–3.4)	59	21	21
Patient was referred to a Centre of Expertise	No	40 (2594)	5.4	1.0 (0.1–6.2)	48	23	29
	Yes	60 (3875)	4.3	0.7 (0.1–4.2)	54	23	23
Characteristics of the diagnosis journey (family and support)							
Family members already diagnosed	No	89 (5797)	4.4	0.8 (0.1–4.5)	53	23	24
	Yes	11 (707)	7.1	1.2 (0.0–10.0)	47	17	35
Financial support	Needs met	28 (1801)	3.8	0.6 (0.1–3.5)	56	23	21
	Needs unmet	72 (4668)	5.1	0.9 (0.1–5.8)	50	23	27
Psychological support	Needs met	27 (1754)	3.8	0.6 (0.0–3.4)	57	22	21
	Needs unmet	73 (4753)	5.1	1.0 (0.1–5.7)	50	23	27

Characteristics of the rare disease and associated symptoms							
Number of body parts affected	1–7	88 (5733)	4.3	0.7 (0.1–4.3)	54	23	23
	8+	22 (774)	7.9	2.3 (0.3–11.0)	39	22	39
Genetic disease	Yes	66 (3632)	5.9	1.2 (0.1–7.4)	47	22	30
	No	34 (1888)	2.6	0.4 (0.1–2.3)	63	21	16
Skin diseases	Yes	25 (1385)	7.6	1.8 (0.2–11.0)	41	22	37
	No	75 (4135)	3.8	0.6 (0.1–3.7)	56	22	22
Gastroenterological diseases	Yes	4 (217)	2.9	0.2 (0.0–1.9)	69	15	16
	No	96 (5303)	4.9	0.8 (0.1–5.3)	52	22	26
Gynaecologic-obstetric diseases	Yes	4 (214)	2.8	0.3 (0.0–3.1)	61	22	16
	No	96 (5306)	4.9	0.8 (0.1–5.3)	52	22	26
Renal diseases	Yes	9 (594)	3.3	0.4 (0.0–2.4)	61	21	18
	No	91 (4926)	5.0	0.8 (0.1–5.5)	51	22	26
Odontological diseases	Yes	2 (116)	12.3	6.9 (0.8–20.2)	26	19	55
	No	98 (5404)	4.6	0.8 (0.1–4.9)	53	22	25
Hepatic diseases	Yes	10 (620)	6.2	0.7 (0.0–7.9)	53	16	31
	No	90 (4900)	4.6	0.8 (0.1–4.9)	52	23	25
Respiratory diseases	Yes	8 (546)	6.7	0.8 (0.0–8.8)	51	18	31
	No	92 (4947)	4.6	0.8 (0.1–4.9)	53	23	25
Neurological diseases	Yes	43 (2802)	4.3	0.8 (0.1–4.5)	52	24	24
	No	57 (2718)	5.3	0.7 (0.1–5.7)	53	20	27
Sudden onset of symptoms requiring urgent care	Yes	45 (2940)	4.8	0.7 (0.1–5.0)	54	21	25
	No	54 (3280)	4.6	0.9 (0.1–5.0)	51	24	25
	Don’t know	4 (287)	5.1	0.9 (0.0–5.7)	50	23	26
Outbreaks (clinical signs or symptoms that come and go)	Yes	56 (3680)	5.4	1 (0.1–6.2)	48	23	28
	No	37 (2417)	3.8	0.6 (0.1–3.3)	57	21	21
	Don’t know	6 (410)	4.1	0.7 (0.0–4.1)	54	24	22
Total		**100 (6507)**	**4.7**	**0.8 (0.1–5.0)**	**52**	**23**	**25**

Distribution: percentage and frequencies of respondents for the categories of each variable (percentages in column-totals may not be equal between variables because of missing values); % < 1, % 1–4, % ≥ 5 = percentage of respondents who experienced a TDT of less than 1 year; 1–4 year; 5 years or more in each category (percentages in row).

*%* Percentage, *N* number of observations (totals are not equal between categories because of missing values), *IQR* Interquartile range.

Bold values refer to the corresponding figures for the whole sample (6507 people): average TDT is 4.7 years, median TDT is 0.8 years (IQR = 0.1-5.0), 52% respondents were diagnosed in less that 1 year, 23% within 1 to 4 years, and 25% in within 5 years or more.

**Table 2:** Main variables associated with diagnosis delays, and their association with patient delays and health system delays.

Variables	Category	Patient delay		Health system delay		Diagnosis delay	
		OR (95% CI)	*P*	OR (95% CI)	*P*	OR (95% CI)	*P*
Sociodemographic characteristics							
Age of the patient at perceived symptom onset	<2	1.92	***	1.17	*NS*	2.26	***
		(1.5–2.4)		(0.9 – 1.5)		(1.8 – 2.8)	
	2–9	1.57	***	1.95	***	3.10	***
		(1.2–2.0)		(1.5–2.5)		(2.4–3.9)	
	10–19	2.70	***	1.71	***	4.74	***
		(2.1–3.5)		(1.3–2.2)		(3.7–6.2)	
	20–29	1.74	***	1.54	***	2.44	***
		(1.4–2.2)		(1.2–2.0)		(1.9–3.1)	
	30–49	1.33	***	1.36	***	1.70	***
		(1.1–1.6)		(1.1–1.7)		(1.4–2.0)	
	50 and more	ref (-)		ref (-)		ref (-)	
Gender of the patient	Male	ref (-)		ref (-)		ref (-)	
	Female	1.13	*	1.27	***	1.22	***
		(1.0–1.3)		(1.1–1.5)		(1.1–1.4)	
Country Group	Eastern, Central and Southern Europe	ref (-)		ref (-)		ref (-)	
	Western Europe	1.23	**	1.89	***	1.95	***
		(1.0–1.5)		(1.5–2.3)		(1.6–2.3)	
	Northern Europe	1.07	*NS*	2.30	***	2.11	***
		(0.9–1.3)		(1.9–2.8)		(1.7–2.5)	
Characteristics of the diagnosis journey (healthcare system)							
Number of healthcare professionals consulted	0–1	ref (-)		ref (-)		ref (-)	
	2–7	0.96	*NS*	2.03	***	1.86	***
		(0.8–1.1)		(1.6–2.5)		(1.5–2.3)	
	8+	0.90	*NS*	6.15	***	5.06	***
		(0.7–1.1)		(4.7–8.0)		(4.1–6.4)	
	No	ref (-)		ref (-)		ref (-)	
Misdiagnosis	Yes	1.04	*NS*	2.72	***	2.42	***
		(0.9–1.2)		(2.4–3.2)		(2.1–2.8)	
	No	ref (-)		ref (-)		ref (-)	
	Yes	0.91	**	1.64	***	1.55	***
Genetic tests		(0.8–1.1)		(1.4–1.9)		(1.3–1.8)	
	Don’t know/not relevant	0.76	**	0.78	*	0.57	***
		(0.6–1.0)		(0.6–1.0)		(0.4–0.7)	
Healthcare professionals reluctant or not sufficiently informed to prescribe genetic tests (declarative)	No	ref (-)		ref (-)		ref (-)	
	Yes	1.0	*NS*	1.65	***	1.54	***
		(0.9–1.2)		(1.4–1.9)		(1.3–1.8)	
	Not relevant	0.93	*NS*	1.16	*	1.07	*NS*
		(0.8–1.1)		(1–1.4)		(0.9–1.2)	
	No	1.09	NS	1.10	NS	1.17	***
The patient was referred to a Centre of Expertise	Yes	(0.97–1.22)		(1.0–1.2)		(1.0–1.3)	
		ref (-)		ref (-)		ref (-)	
Characteristics of the diagnosis journey (family and support)							
Family members already diagnosed	No	ref (-)		ref (-)		ref (-)	
	Yes	1.37	***	1.01	*NS*	1.37	***
		(1.1–1.7)		(0.8–1.2)		(1.1–1.6)	
Financial support	Needs met	ref (-)		ref (-)		ref (-)	
	Needs unmet	1.06	*NS*	1.12	*NS*	1.17	**
		(0.9–1.2)		(1.0–1.3)		(1.0–1.3)	
	Needs met	ref (-)		ref (-)		ref (-)	
Psychological support	Needs unmet	1.10	*NS*	1.20	**	1.33	***
		(1.0–1.2)		(1.0–1.4)		(1.2–1.5)	
Characteristics of the rare diseases and symptoms							
Number of body parts affected	1–7	ref (-)		ref (-)		ref (-)	
	8+	0.94	*NS*	1.26	**	1.10	*NS*
		(0.8–1.1)		(1.0–1.5)		(0.9–1.3)	
	No	ref (-)		ref (-)		ref (-)	
Genetic disease	Yes	0.89	*NS*	1.75	***	1.34	***
		(0.8–1.0)		(1.5–2.0)		(1.1–1.6)	
	No	ref (-)		ref (-)		ref (-)	
Gastroenterological disease	Yes	0.64	***	0.73	*	0.52	***
		(0.5–0.9)		(0.5–1.1)		(0.4–0.7)	
	No	ref (-)		ref (-)		ref (-)	
Gynaecologic-obstetric disease	Yes	0.94	*NS*	0.65	**	0.63	***
		(0.7–1.3)		(0.5–0.9)		(0.5–0.8)	
	No	ref (-)		ref (-)		ref (-)	
Renal disease	Yes	0.90	*NS*	0.52	***	0.54	***
		(0.7–1.1)		(0.4–0.6)		(0.4–0.7)	
	No	ref (-)		ref (-)		ref (-)	
Neurological disease	Yes	1.15	*NS*	0.90	*NS*	1.00	*NS*
Outbreaks (clinical signs or symptoms that come and go)		(1.02–1.3)		(0.8–1.0)		(0.9–1.1)	
	No	ref (-)		ref (-)		ref (-)	
	Yes	0.96	*NS*	1.18	**	1.16	**
		(0.8–1.1)		(1.0–1.3)		(1.0–1.3)	
	Don’t know	1.09	*NS*	0.95	*NS*	1.08	*NS*
		(0.8–1.4)		(0.7–1.2)		(0.8–1.4)	
Sudden onset of symptoms requiring urgent care	No	ref (-)		ref (-)		ref (-)	
	Yes	0.72	***	0.79	***	0.88	***
		(0.6–0.8)		(0.7–0.9)		(0.7–0.8)	
	Don’t know	0.86	*NS*	0.93	*NS*	0.91	*NS*
		(0.64–1.1)		(0.7–1.2)		(0.7–1.2)	
Constant cut1		0.39	***	43.63	***	50.2	***
		(0.3–0.5)		(28.9–65.9)		(34.3–73.5)	
Constant cut2		5.5	***	157.3	***	172.19	***
		(3.9–7.7)		(102.8–240.5)		(116.4–254.8)	
Observations		4617		4617		5220	
R2		0.02		13.94		13.97	

All models are adjusted for ‘Not in contact with other patients because of accessibility issues’, ‘Odontological diseases’, ‘Hepatic diseases’ and ‘Respiratory diseases’. Constant Cut or “cut point” values are defined by the ratio of cases below the cut point to cases above the cut point.

*OR* Odd Ratios, *CI* Confidence Interval, *Ref (-)* Reference group, *RD* rare disease, *NS* non-significant, *R²* Coefficient of determination.

***p < 0.01, **p < 0.05, *p < 0.1.

The patient’s age at symptom onset was calculated based on the patients’ declared birth date (entered by family members when respondents were not patients themselves) and date of symptom onset. 11% (707/6504) of respondents reported that a family member was already diagnosed with the same disease before the patient was diagnosed, 55% (2506/6507) of the patients had genetic tests as part of their diagnosis search, and 23% (1493/6507) of the respondents had perceived reluctance from doctors to prescribe a genetic test. Of note, 90% (699/781) of the patients who had a genetic test despite doctors’ hesitation were diagnosed with a genetic condition. Among the 3632 patients with a genetic RD, 26% (943/3632) perceived hesitation to be prescribed a genetic test by healthcare professionals and 75% (2724/3632) had access to a genetic test. Among the 2423 patients who had genetic tests and were diagnosed after 2010, 26% (623) said that they had single-gene sequencing, 34% (819) gene-panel sequencing, 24% (591) Genome Sequencing (GS) or Exome Sequencing (ES) and 25% (597) that they did not know which test was conducted.

**Fig. 2:**
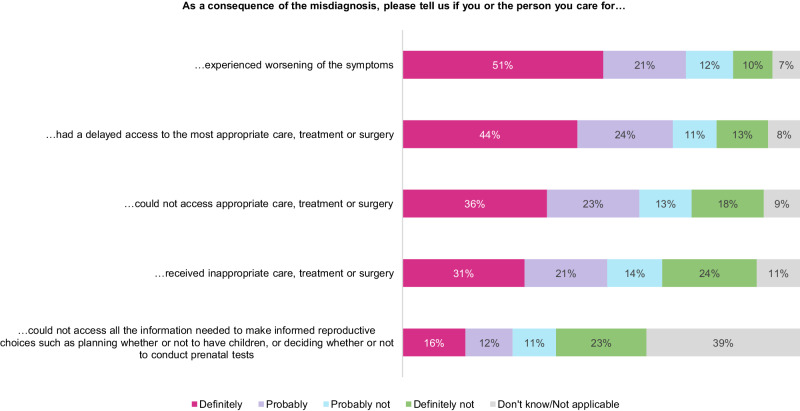
Consequences of misdiagnosis. Respondents who said that their RD, or the RD of the person they care for, have been misdiagnosed at least once (*n* = 4756), i.e. that the symptoms of the RD have been attributed to another physical disease, or that symptoms were neglected, not taken seriously or considered as psychological.

22% (1451/6507) of the patients consulted at least 8 healthcare professionals when searching for a diagnosis, 73% (4756/6507) were misdiagnosed at least one time, *i.e*., their symptoms have been attributed to another physical disease, neglected, not taken seriously or considered as psychological. Respondents saw an impact of the misdiagnosis on access to the most appropriate care, treatment, or surgery: access was delayed for 68% of them (3254/4756), prevented for 59% (2819/4756), or inappropriate for 52% (2477/4756) (Fig. 2). Many RD patients diagnosed within a year have also encountered difficulties, as 28% of them (956/3383) consulted 5 healthcare professionals or more, and 62% (2110/3383) were misdiagnosed at least once.

**Fig. 3:**
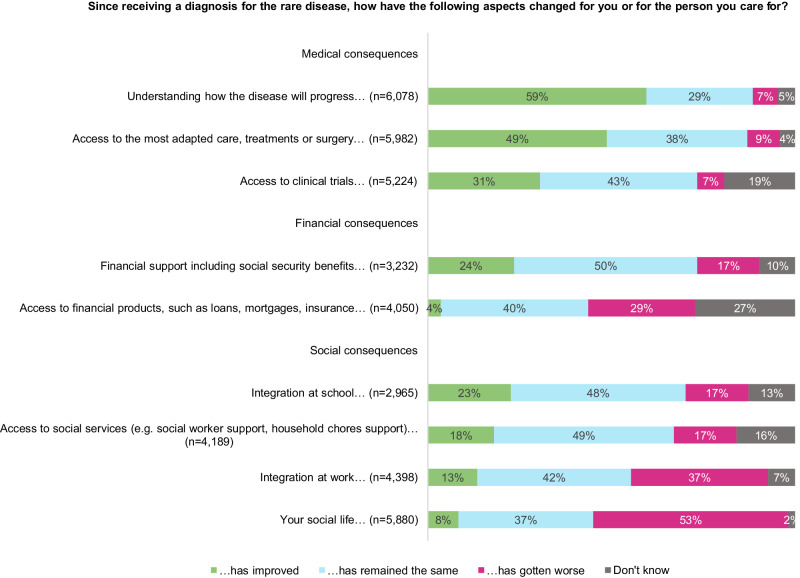
Post-diagnosis changes. Responses ‘not relevant’ were removed: totals are not equal between categories because of varying number of responses ‘not relevant’ and missing values.

When asked about post-diagnosis changes (Fig. 3), most respondents said that their understanding of disease progression and their access to the most adapted care, treatment or surgery had improved (respectively 59% and 49%); most of them said that their access to financial support and products, to social services, to clinical trials, as well as integration at work or at school remained unchanged (between 40% and 50%); 53% of the respondents said that their social life had worsened since the diagnosis.

### Total diagnosis time and associated difficulties

In Europe, the average TDT took 4.7 years. 50% of PLWRD waited at least 9 months (median) for a confirmed diagnosis after symptom onset, and 25% waited more than 5 years (third quartile). Diagnostic delays mainly stemmed from the health system: the average time from symptom onset to first medical contact was approximately 5 months, while the average time from first medical contact to confirmed diagnosis was 4.3 years.

Patients who consulted more than 8 healthcare professionals as part of their diagnosis journey (OR = 5.06, 95% CI: 4.1–6.4) and those who have been misdiagnosed at least one time (OR = 2.42, 95% CI:2.1–2.8) were more likely to have experienced diagnostic delays (Table 2).

### Main determinants of diagnostic delays

The primary determinant of the diagnostic delay was the age of the patient at perceived symptom onset: patients who were children (OR = 3.10; 95% CI:2.4–3.9), adolescents (OR = 4.74; 95% CI: 3.7–6.2) and young adults (OR = 2.44; 95% CI:1.9–3.1) at symptom onset were more likely to have a diagnostic odyssey than patients who were older at symptom onset, and this comes from a higher risk of PD and of HSD. Patients in Eastern, Central and Southern Europe had a lower risk of experiencing diagnostic delays than those living in Western Europe (OR = 1.95, 95% CI:1.6–2.3) and Northern Europe (OR = 2.11, 95% CI:1.7–2.5). Diagnostic delays were more likely to occur among women (OR = 1.22; 95% CI:1.1–1.4), and gender had a more substantial impact on HSD (OR = 1.27; 95% CI: 1.1–1.5) than on PD (OR = 1.13; 95% CI: 1.0–1.3).

Being referred to a Centre of Expertise contributed to limiting the risk of diagnostic delays (OR = 1.17, 95% CI:1.0–1.3) for the 60% (3875/6469) patients who were referred to such specialised hospital units (Table 1). Respondents whose needs for financial support (OR = 1.17; 95% CI: 1.0–1.3) and psychological support (OR = 1.33; 95% CI: 1.2–1.5) were met during their diagnosis search had fewer chances of experiencing diagnostic delays, while limited access to psychological support was associated with an increased risk of HSD (OR = 1,20; 95% CI: 1.0–1.4).

Unexpectedly, patients who had a family member already diagnosed with the same RD had more risk to experience diagnostic delays (OR = 1.37, 95% CI: 1.1–1.6), whether the RD was genetic (OR = 1.23; 95% CI: 1.0–1.5, *p* = 0.05) or not (OR = 2.14; 95% CI: 1.3–3.6; *p* < 0.01). This delay mostly stemmed from PD. Among respondents who declared a family history of the same disease, 25% (176/707) were living with HHT and 5% (33/707) with EDS: after controlling the disease overrepresentation by only considering respondents who were not living with HHT or EDS, there was no significant difference between patients with and without a family history, not even among the other genetic diseases.

The chances of experiencing diagnostic delays were higher for people living with genetic RD (OR = 1.34, 95% CI:1.1–1.6) compared to those living with non-genetic RD. Patients who eventually underwent genetic tests were more likely to have experienced diagnostic delays (OR = 1.55, 95% CI: 1.3–1.8). Complementary analysis conducted on the 2376 respondents living with genetic RD diagnosed after 2010 showed that the risk of diagnostic delays did not depend on the type of genetic test conducted (single-gene sequencing, gene-panel sequencing, GS or ES).

Regarding the type of diseases and organs involved, we found that people with gastroenterological, gynaecological or kidney RD were less likely to experience diagnostic delays, unlike people with skin diseases. Diseases manifesting through symptoms outbreaks have more risks of diagnostic delays (OR = 1.16; 06% CI: 1.0–1.03), mostly due to HSD, while those with sudden onset of symptoms requiring emergency care have fewer risks of diagnostic delays (OR = 0.88; 95% CI: 0.7–0.9), from lower risks of both PD and HSD.

## Discussion

### The diagnostic odyssey: a long and difficult journey for most Europeans living with a diagnosed RD

This article is the first to investigate the TDT for people living with such a broad scope of RD in Europe and to identify the main determinants of their diagnostic delays. Our findings demonstrate that even if individual RD differ, PLWRD experience common challenges, the first being obtaining a diagnosis. This diagnosis search often takes years: 56% of respondents were diagnosed more than six months after coming to medical attention (a key system goal from the Rare 2030 foresight study [2]), and 48% waited more than one year (a key system goal from IRDiRC [3]), which is consistent with existing literature in Europe [4], Spain [5, 6], France [7] and the UK [8].

To better understand how to reduce diagnostic delays, we distinguished PD and HSD, and we found that the time spent searching for a diagnosis in the health system accounts for 90% of the average TDT. Within the health systems, barriers to faster diagnosis identified in the literature include physicians’ lack of understanding and awareness of RD, long waiting times to be referred to or consult a specialist, and lack of access to adequate diagnostic tools [9]. These limitations might also explain the propensity for patients with the highest risk of diagnostic delays to consult multiple health professionals.

Like other studies, our survey confirms that the diagnostic odyssey of PLWRD was characterised by its duration and associated difficulties as it often included multiple visits to healthcare professionals and numerous misdiagnoses (which ultimately lead to inappropriate care, treatments or surgeries or to lack of specialised care [4–12]), even when patients were diagnosed within a year. It also shows that diagnosis often improved access to care, treatment or surgery for PLWRD. However, access to financial and social support was seldom improved after diagnosis, most probably because of the lack of social acceptance of RD and their impacts, which hinders access to adequate social benefits and independent living support for PLWRD and their family. For many respondents, social life had even worsened post-diagnosis, probably due to more time spent every day in tasks related to care and care coordination [16].

### Main determinants of the diagnostic delays for Europeans living with an RD

In our study, the patient’s age at symptom onset was the primary determinant of diagnostic delays. People who were children, adolescents and young adults at symptom onset were more likely to experience diagnostic delays because of higher risks of both PD and HSD. A 2016 survey of 844 French people living with 22 RD [7] also found that hospital referral delays and diagnostic delays were longer when patients were 2–18 years of age at symptom onset, than when symptoms appeared in infancy or adulthood. In the literature on rare and common diseases, on the contrary, parents are generally described as weighing in positively in the diagnosis process of infants and children, initiating or coordinating their care [17–19]. Most of the existing literature on PD in paediatric RD focuses on paediatric cancers: a case study on two adolescents with genital tumours [20] showed how embarrassment and fear experienced by adolescents led to extended PD. Another study on the pre-diagnosis cancer experience of adolescents and young adults [21] showed that diagnostic delays could come from patient, doctor and health system delays, with a common reaction from adults that young people do not get cancer. This experience was described either as “normalisation/adaptation”, where symptoms were usually appraised and minimised by the patients or the adults surrounding them (family members or health professionals) or as “acute symptoms/immediate threat” when there was little or no possibility of normalisation, minimisation or adaptation. Testimonies from parents of children living with an RD show that most of the diagnosis search relies on them, including time constraints associated with becoming experts in their child’s RD, navigating the healthcare system while caring for their family, and managing the emotional process of accepting the consequences of the RD on their child and on their family [22–26]. This could explain why accessing financial and psychological support decreases the risks of diagnostic delays.

Once we controlled for overrepresentation of patients with HHT and EDS among respondents who declared a family history with their disease, we found no significant difference between patients with and without a family history of the RD, not even for the remaining genetic disorders. An Italian retrospective study on HHT found that compared to patients who were the first to receive an HHT diagnosis in their family, patients with a family history of HHT had shorter TDT (29.1 vs 22.6 years; *p* < 0.02) and did not have significant longer PD (15.5 vs 14.8 years; *p* = 0.68) [27]. In our study, since the delay stemmed from patients, chances are that those patients and their families postponed or refused to confirm the diagnosis of the RD, for example because they assumed that they were living with the same RD as their family member, because symptoms were not very severe at that time, due to a form of denial, or by fear of paying more health insurance fees. As for the HSD, our results showed that in health systems, patients with a family history of the disease may have encountered similar difficulties as patients without a family history.

We found that women had a higher risk of diagnostic delays, especially after they entered the health system, as reflected in a study showing that in France, each step of the diagnosis journey was longer for women than for men living with 22 different RD, even when controlling for gender-related prevalence by only analysing RD that are similarly prevalent in both sexes [7]. In the general population, a study examining health data for almost 7 million men and women in the Danish healthcare system for over 21 years also showed that women were diagnosed later than men in more than 700 conditions [28]. A 2020 scoping review showed that gender disparities in specialised healthcare could be reduced and even eliminated if clinicians’ adherence to guidelines increased and that there was a lack of studies addressing this problem in primary healthcare, where the impact of narrowing gender bias could be more significant [29]. However, further analysis would be needed to see if there are disease-related or country-related phenomena regarding gender disparities in healthcare for RD, as found in some studies [30–32].

Further research would be needed to determine why Northern and Western Europeans had a higher risk of diagnostic delays. Differences could come from higher investments in diagnostic services among Northern and Western European countries [33], allowing health systems to be better equipped to end long diagnosis journeys or to diagnose a broader range of RD. Likewise, PLWRD in Eastern, Central and Southern European countries (which have a lower GDP and investment in health systems), may be diagnosed either fast or not at all, remaining undiagnosed.

The use of genetic testing undoubtedly improves the diagnosis of genetic diseases. Although our findings point out a positive association between having a genetic test or a genetic disorder and experiencing diagnostic delays, they mostly reflect the difficulties in accessing genetic tests and the long lag time before obtaining results, calling for policies and actions to improve availability and affordability of genetic testing and counselling for PLWRD.

People living with very rare diseases did not have significantly higher diagnostic delays, meaning that regarding diagnosis and when considering all RD, “rare is rare”. The size of the city where respondents lived and their education level were not found to be predictors of diagnostic delays, which is coherent with results from similar Spanish [5, 6, 11] and French studies [7] on RDs, but significantly different from studies on common diseases, showing that health determinants for RD substantially differ from those for common diseases [34].

### Shortening the diagnosis journey of PLWRD

If PLWRD encounter common challenges when searching a diagnosis, some differences observed in our study show that the measure of the time to diagnosis for all PLWRD cannot be used as a stand-alone indicator to monitor RD diagnosis at a national and a European level as (i) TDT may increase when RD are better known and better managed in national healthcare systems, or when new diagnosis technologies allow to end more diagnostic odysseys and (ii) this measure does not include RD patients who are still waiting for a confirmed diagnosis. However, when coupled with a better understanding of the main determinants of diagnostic delays, TDT is a robust indicator to inform policies and actions that could be taken to improve access to diagnosis for PLWRD.

While PD only accounts for 10% of the average TDT, it is more critical for children, adolescents and young adults that could benefit from raising knowledge of RD in the general population. Improved recognition of PLWRD in societies could also help prevent fear of discrimination and popularise online diagnostic tools accessible to the broader public.

Improving knowledge of RD among healthcare professionals, especially in primary care, should improve the identification of uncommon patterns, encourage healthcare professionals to think outside the box of common symptoms and diseases encountered daily, and provide them with adequate information on existing Centres of Expertise and networks at a national and European level (European Reference Networks), where they could refer patients with a suspected RD. Our results showed that the sudden onset of symptoms, probably because of their urgent nature, is quickly acknowledged and investigated, leading to a shorter diagnostic journey. The challenge remains in more silent or inconsistent manifestations. Therefore, courses on RD in medical students’ curricula and continuous training of healthcare professionals could be enforced at a national level to raise awareness on RD common characteristics, manifestations and prevalence (3.5%–5.9% of the population, 30 million people in Europe), while considering barriers such as gender-based bias in RD diagnosis.

### Strengths and limitations

While this study is the first to investigate the TDT for people living with such a broad scope of RD in 41 European countries, it has limitations inherent to any declarative retrospective survey on RD: (i) by lack of epidemiological studies on RD, recruitment bias cannot be estimated precisely; (ii) this study does not allow to monitor the evolution of TDT over time as this measure is subject to too many structural effects and does not fully capture the changing situation regarding genetic tests, which are increasingly realised at first intention and lower cost, at least in some European countries.

## Conclusion

Diagnosis delays were not impacted by respondents’ level of education and city size or by the point prevalence of their RD, showing that the unmet needs of PLWRD and their families in Europe should be addressed by tackling the specificity of RD determinants at national and European levels.

HSD accounted for most of the TDT and could be reduced by improving awareness of all RD among primary care professionals, improving referral to Centres of Expertise and reducing gender disparities in primary care and specialised care. Improved public awareness of RD could contribute to lowering the risks of PD encountered by children and adolescents. Access to diagnosis improved access to healthcare but seldom improved access to financial and social support, calling for a better social acceptance of RD and their impacts, and a more holistic care approach for PLWRD.

New diagnostic technologies using the most recent advances in genetics and omics create a home for improved and curtailed access to diagnosis for people living with a genetic RD, including as part of newborn screening programmes. Still, these technologies may come with new difficulties such as access to appropriate genetic counselling, communication within the family, or granting faster access to test results across Europe.

### Supplementary information

Additional File 1

Additional File 2

Additional File 3

Additional File 4

Additional File 5

Additional File 6

Video Summary

## Data Availability

The survey data is not available in a repository as, while pseudonymised, it includes information that could make the respondents identifiable for instance by crossing the country of residence and the name of ultra-rare diseases. Collective results are available upon request to the corresponding author or to rare.barometer@eurordis.org. Members of EURORDIS-Rare Diseases Europe (eurordis.org/who-we-are/our-members) and of Rare Diseases International (rarediseasesinternational.org/members-list) have access to collective results for their community (e.g. all RD in one country; one RD in Europe or worldwide; one RD or a group of RD in one country;…) and should be contacted directly.
