# Supplementary material for: Time to diagnosis and determinants of diagnostic delays of people living with a rare disease: results of a Rare Barometer retrospective patient survey
Source: Eur J Hum Genet. 2024 May 16;32(9):1116–26. doi: 10.1038/s41431-024-01604-z (PMC11369105; doi:10.1038/s41431-024-01604-z)
Supplement: Supplementary file 1 — Additional File 1 [file 41431_2024_1604_MOESM1_ESM.docx]

**Additional file 1: List of rare diseases with at least 20 respondents**

| **Name of the rare disease (declarative)** | **Survey respondents % (n)** |
| --- | --- |
| Hereditary hemorrhagic telangiectasia (HHT) | 4.5% (296) |
| Ehlers-Danlos syndrome (EDS) | 4.6% (297) |
| Hypermobile Ehlers-Danlos syndrome | 2.9% (188) |
| Classical Ehlers-Danlos syndrome | 1.0% (68) |
| Classical-like Ehlers-Danlos syndrome type 1 | 0.2% (14) |
| Ehlers-Danlos syndrome | 0.2% (14) |
| Vascular Ehlers-Danlos syndrome | 0.2% (13) |
| Sarcoidosis | 2.1% (136) |
| Williams syndrome | 1.6% (101) |
| Myasthenia gravis | 1.4% (92) |
| Cystic fibrosis | 1.4% (90) |
| Systemic sclerosis | 1.3% (84) |
| Addison disease | 1.0% (64) |
| Tuberous sclerosis complex | 1.0% (63) |
| Neurofibromatosis type 1 | 0.9% (60) |
| 22q11.2 deletion syndrome | 0.8% (55) |
| Interstitial cystitis | 0.8% (55) |
| Chronic inflammatory demyelinating polyneuropathy | 0.8% (52) |
| Acute inflammatory demyelinating polyradiculoneuropathy | 0.7% (47) |
| Rett syndrome | 0.7% (45) |
| Perineural cyst | 0.6% (42) |
| Primary sclerosing cholangitis | 0.6% (40) |
| Fragile X syndrome | 0.6% (37) |
| Granulomatosis with polyangiitis | 0.6% (37) |
| Wilson disease | 0.6% (37) |
| Duchenne muscular dystrophy | 0.5% (35) |
| Prader-Willi syndrome | 0.5% (35) |
| Marfan syndrome | 0.5% (34) |
| Arnold-Chiari malformation type I | 0.5% (31) |
| Autosomal systemic lupus erythematosus | 0.5% (31) |
| Primary lymphedema | 0.5% (30) |
| Scleroderma | 0.5% (30) |
| Fabry disease | 0.4% (29) |
| Angelman syndrome | 0.4% (27) |
| Behçet disease | 0.4% (27) |
| Neonatal antiphospholipid syndrome | 0.4% (27) |
| Common variable immunodeficiency | 0.4% (26) |
| Diffuse cutaneous systemic sclerosis | 0.4% (25) |
| Lupus erythematosus panniculitis | 0.4% (25) |
| Non-acquired panhypopituitarism | 0.4% (25) |
| Desmoid tumor | 0.4% (24) |
| Autoimmune hepatitis | 0.3% (22) |
| Hereditary spastic paraplegia | 0.3% (22) |
| Idiopathic pulmonary fibrosis | 0.3% (22) |
| Narcolepsy type 1 | 0.3% (21) |
| Phenylketonuria | 0.3% (21) |
| Pseudomyxoma peritonei | 0.3% (20) |
| **TOTAL** | 100.0% (6507) |
