# Supplementary material for: Time to diagnosis and determinants of diagnostic delays of people living with a rare disease: results of a Rare Barometer retrospective patient survey
Source: Eur J Hum Genet. 2024 May 16;32(9):1116–26. doi: 10.1038/s41431-024-01604-z (PMC11369105; doi:10.1038/s41431-024-01604-z)
Supplement: Supplementary file 2 — Additional File 2 [file 41431_2024_1604_MOESM2_ESM.docx]

**Additional file 2: Orphanet classification based on organs or systems affected, and on the genetic nature of the RD**

| **Orphanet classification**  *One disease can be classified in several categories.* | **Survey respondents % (n)** |
| --- | --- |
| Genetic diseases | 56 (3,632) |
| Neurological diseases | 44 (2,884) |
| Developmental anomalies during embryogenesis | 34 (2,189) |
| Transplant related diseases | 27 (1,752) |
| Skin diseases | 21 (1,388) |
| Ophthalmic diseases | 19 (1,253) |
| Circulatory system diseases | 14 (907) |
| Endocrine diseases | 12 (771) |
| Hepatic diseases | 10 (620) |
| Neoplastic diseases | 9 (610) |
| Renal diseases | 9 (595) |
| Inborn errors of metabolism | 9 (563) |
| Respiratory diseases | 9 (554) |
| Bone diseases | 8 (539) |
| Cardiac diseases | 8 (516) |
| Systemic or rheumatologic diseases | 7 (429) |
| Infertility | 5 (315) |
| Haematological diseases | 4 (282) |
| Cardiac malformations | 3 (219) |
| Gastroenterological diseases | 3 (218) |
| Gynaecologic/obstetric diseases | 3 (214) |
| Immunological diseases | 3 (203) |
| Otorhinolaryngologic diseases | 3 (149) |
| Abdominal surgical diseases | 2 (134) |
| Odontological diseases | 2 (116) |
| Urogenital diseases | 2 (107) |
| Surgical maxillo-facial diseases | 1 (80) |
| Other | 0 (17) |
| **TOTAL** | **100 (6,507)** |
