# Supplementary material for: Time to diagnosis and determinants of diagnostic delays of people living with a rare disease: results of a Rare Barometer retrospective patient survey
Source: Eur J Hum Genet. 2024 May 16;32(9):1116–26. doi: 10.1038/s41431-024-01604-z (PMC11369105; doi:10.1038/s41431-024-01604-z)
Supplement: Supplementary file 3 — Additional File 3 [file 41431_2024_1604_MOESM3_ESM.docx]

**Additional file 3: Characteristics of countries and country groups**

|  | **Respondents** | |  | **Population** | |  | **Macroeconomic indicators** | | | | |
| --- | --- | --- | --- | --- | --- | --- | --- | --- | --- | --- | --- |
| **Country** | **N** | **%** |  | **N** | **%** |  | **Population density^1^** | **GDP per capita^2^** | **Government health expenditure as part of GDP^3^** | **Government health expenditure per capita^4^** | **Government health expenditure total^5^** |
| **Eastern & Southern Europe** | **988** | **15.2%** |  | **408,955,518** | **49.6%** |  | **82*** | **13,861*** | **6.9%*** | **1,348*** | **8,028*** |
| Andorra | 2 | 0.0% |  | 77,265 | 0.0% |  | 164 | 34,588 | 6.8% | 2,450 | 142 |
| Belarus | 0 | 0.0% |  | 9,379,952 | 1.1% |  | 46 | 6,235 | 5.5% | 757 | 2,430 |
| Bosnia and Herzegovina | 15 | 0.2% |  | 3,280,815 | 0.4% |  | 64 | 5,438 | 8.9% | 956 | 1,220 |
| Bulgaria | 64 | 1.0% |  | 6,934,015 | 0.8% |  | 64 | 7,921 | 7.3% | 997 | 2,798 |
| Croatia | 114 | 1.8% |  | 4,047,680 | 0.5% |  | 72 | 12,985 | 6.8% | 1,656 | 3,334 |
| Cyprus | 38 | 0.6% |  | 1,207,361 | 0.1% |  | 131 | 26,503 | 6.8% | 1,214 | 699 |
| Czech Republic | 117 | 1.8% |  | 10,697,858 | 1.3% |  | 139 | 18,985 | 7.5% | 2,646 | 15,248 |
| Estonia | 2 | 0.0% |  | 1,329,479 | 0.2% |  | 31 | 19,767 | 6.7% | 1,792 | 1,475 |
| Greece | 120 | 1.8% |  | 10,700,556 | 1.3% |  | 83 | 17,324 | 8.0% | 1,219 | 8,288 |
| Hungary | 105 | 1.6% |  | 9,750,149 | 1.2% |  | 107 | 14,401 | 6.5% | 1,449 | 7,115 |
| Latvia | 45 | 0.7% |  | 1,900,449 | 0.2% |  | 31 | 15,560 | 6.2% | 1,134 | 1,234 |
| Lithuania | 13 | 0.2% |  | 2,794,885 | 0.3% |  | 45 | 17,214 | 6.5% | 1,612 | 2,258 |
| Macedonia | 4 | 0.1% |  | 2,072,531 | 0.3% |  | 82 | 5,067 | 6.6% | 646 | 457 |
| Montenegro | 1 | 0.0% |  | 621,306 | 0.1% |  | 46 | 6,516 | 8.3% | 1,095 | 265 |
| Poland | 46 | 0.7% |  | 37,899,070 | 4.6% |  | 124 | 14,661 | 6.3% | 1,438 | 25,649 |
| Romania | 83 | 1.3% |  | 19,257,520 | 2.3% |  | 84 | 10,865 | 5.6% | 1,333 | 10,622 |
| Russia | 27 | 0.4% |  | 144,073,139 | 17.5% |  | 9 | 9,711 | 5.4% | 934 | 53,016 |
| Serbia | 27 | 0.4% |  | 6,899,126 | 0.8% |  | 79 | 6,549 | 8.5% | 924 | 2,501 |
| Slovakia | 42 | 0.6% |  | 5,458,827 | 0.7% |  | 114 | 17,361 | 6.7% | 1,674 | 5,447 |
| Slovenia | 24 | 0.4% |  | 2,102,419 | 0.3% |  | 104 | 22,899 | 8.3% | 2,415 | 3,148 |
| Turkey | 62 | 1.0% |  | 84,339,067 | 10.2% |  | 110 | 12,039 | 4.1% | 892 | 24,079 |
| Ukraine | 37 | 0.6% |  | 44,132,049 | 5.4% |  | 76 | 2,350 | 7.5% | 421 | 5,200 |
| **Northern Europe** | **2,019** | **31.0%** |  | **62,577,493** | **7.6%** |  | **303*** | **57,677*** | **9.0%*** | **4,467*** | **25,775*** |
| Belgium | 574 | 8.8% |  | 11,544,241 | 1.4% |  | 381 | 40,425 | 10.8% | 4,366 | 43,327 |
| Denmark | 240 | 3.7% |  | 5,831,404 | 0.7% |  | 146 | 56,202 | 10.1% | 4,865 | 28,755 |
| Finland | 321 | 4.9% |  | 5,529,543 | 0.7% |  | 18 | 45,010 | 9.0% | 3,527 | 18,852 |
| Iceland | 4 | 0.1% |  | 366,463 | 0.0% |  | 4 | 53,188 | 8.4% | 4,374 | 1,683 |
| Ireland | 60 | 0.9% |  | 4,985,674 | 0.6% |  | 72 | 78,733 | 6.9% | 4,351 | 19,200 |
| Luxembourg | 76 | 1.2% |  | 630,419 | 0.1% |  | 245 | 104,879 | 5.3% | 5,455 | 3,120 |
| Malta | 11 | 0.2% |  | 515,332 | 0.1% |  | 1,610 | 25,427 | 8.8% | 2,616 | 807 |
| Netherlands | 333 | 5.1% |  | 17,441,500 | 2.1% |  | 518 | 46,345 | 10.0% | 3,952 | 58,903 |
| Norway | 104 | 1.6% |  | 5,379,475 | 0.7% |  | 15 | 75,017 | 10.0% | 6,007 | 34,543 |
| Sweden | 296 | 4.5% |  | 10,353,442 | 1.3% |  | 25 | 51,542 | 10.9% | 5,152 | 48,560 |
| **Western Europe** | **3,357** | **51.6%** |  | **352,323,368** | **42.8%** |  | **40,421*** | **10.2%** | **3,410*** | **134,329*** | **40,421*** |
| Austria | 62 | 1.0% |  | 8,916,864 | 1.1% |  | 108 | 43,346 | 10.3% | 4,281 | 32,867 |
| France | 622 | 9.6% |  | 67,379,908 | 8.2% |  | 123 | 35,786 | 11.2% | 3,964 | 227,593 |
| Germany | 775 | 11.9% |  | 83,160,871 | 10.1% |  | 238 | 41,315 | 11.5% | 5,042 | 341,042 |
| Italy | 705 | 10.8% |  | 59,449,527 | 7.2% |  | 201 | 29,358 | 8.7% | 2,847 | 128,066 |
| Liechtenstein | 1 | 0.0% |  | 38,137 | 0.0% |  | 238 |  |  |  |  |
| Portugal | 108 | 1.7% |  | 10,297,081 | 1.2% |  | 112 | 19,772 | 9.4% | 2,009 | 13,453 |
| Spain | 652 | 10.0% |  | 47,363,419 | 5.7% |  | 95 | 24,939 | 9.0% | 2,662 | 86,355 |
| Switzerland | 89 | 1.4% |  | 8,636,561 | 1.0% |  | 219 | 85,685 | 11.4% | 2,637 | 25,710 |
| United Kingdom | 343 | 5.3% |  | 67,081,000 | 8.1% |  | 277 | 43,166 | 9.9% | 3,834 | 219,545 |
| Nonresponse | **143** | **2.2%** |  | / | / |  | / | / | / | / | / |
| **TOTAL** | **6,507** | **100.0%** |  | **823,856,379** | **100.0%** |  |  |  |  |  |  |
| ^1^Population density: people per km² (2020, Food and Agriculture Organisation and World Bank Population); ^2^GDP: Gross Domestic Product per capita, 2020 (constant 2015 US$) – World Bank; ^3^Current health expenditure, 2018 (% of GDP) – World Bank; ^4^Domestic general government health expenditure per capita, PPP (current international $), 2018 – World Bank; ^5^Domestic General Government Health Expenditure (GGHE-D) 2018 (million constant (2019) US$) – World Health Organisation; *mean value for the group of countries | | | | | | | | | | | |
