# Supplementary material for: Time to diagnosis and determinants of diagnostic delays of people living with a rare disease: results of a Rare Barometer retrospective patient survey
Source: Eur J Hum Genet. 2024 May 16;32(9):1116–26. doi: 10.1038/s41431-024-01604-z (PMC11369105; doi:10.1038/s41431-024-01604-z)
Supplement: Supplementary file 4 — Additional File 4 [file 41431_2024_1604_MOESM4_ESM.docx]

**Additional file 4: Results of the Kruskal Wallis tests between median TDT and the variables included in the ordinal logistical regressions**

| **Variables** | **chi2** | **p-value** |
| --- | --- | --- |
| **SOCIODEMOGRAPHIC CHARACTERISTICS** | | |
| Age of the patient at perceived symptom onset | 404.844 | 0.0001 |
| Gender of the patient | 39.098 | 0.0001 |
| Country group | 28.356 | 0.0001 |
| **CHARACTERISTICS OF THE DIAGNOSIS JOURNEY (HEALTHCARE SYSTEM)** | | |
| Number of healthcare professionals consulted | 693.249 | 0.0001 |
| Misdiagnosis | 450.761 | 0.0001 |
| Genetic tests | 129.731 | 0.0001 |
| Healthcare professionals were reluctant or not sufficiently informed to prescribe genetic tests (declarative) | 230.671 | 0.0001 |
| The patient was referred to a Centre of Expertise | 31.301 | 0.0001 |
| **CHARACTERISTICS OF THE DIAGNOSIS JOURNEY (FAMILY AND SUPPORT)** | | |
| Family members already diagnosed | 8.676 | 0.0032 |
| Financial support | 24.923 | 0.0001 |
| Psychological support | 37.601 | 0.0001 |
| **CHARACTERISTICS OF THE RARE DISEASE AND ASSOCIATED SYMPTOMS** | | |
| Number of body parts affected | 84.727 | 0.0001 |
| Genetic disease | 123.975 | 0.0001 |
| Skin diseases | 121.883 | 0.0001 |
| Gastroenterological diseases | 32.464 | 0.0001 |
| Gynaecological diseases | 13.923 | 0.0002 |
| Renal diseases | 23.473 | 0.0001 |
| Odontologic diseases | 47.234 | 0.0001 |
| Neurological diseases | 0.045 | 0.8321 |
| Sudden onset of symptoms | 4.675 | 0.0966 |
| Outbreaks | 65.564 | 0.0001 |

*Kruskal–Wallis equality-of-populations rank test*
