# Supplementary material for: Time to diagnosis and determinants of diagnostic delays of people living with a rare disease: results of a Rare Barometer retrospective patient survey
Source: Eur J Hum Genet. 2024 May 16;32(9):1116–26. doi: 10.1038/s41431-024-01604-z (PMC11369105; doi:10.1038/s41431-024-01604-z)
Supplement: Supplementary file 5 — Additional File 5 [file 41431_2024_1604_MOESM5_ESM.docx]

**Additional file 5: Methodological considerations on time-related variables included in the ordinal logistic regressions**

Among the time-related variables that could be calculated based on respondents’ answers, only ‘Age of the patient at perceived symptom onset’ was considered, in this paper, as a determinant of the Total Diagnostic Time (TDT). Three time-related variables were removed from the logistical model presented in **Table 2** as they were subject to too many structural effects:

- age of the patient at the time of the study, present in **Table 1** but not in **Table 2**.
- date of the confirmed diagnosis, present in **Table 1** but not in **Table 2**.
- age of the patient at confirmed diagnosis.

Those choices are explained below, for each of those time-related variables.

1. **Age of the patient at the time of the study**

**Table 1** shows that both the mean and the median TDT increase with the age of the patient at the time of the study, which in our opinion reflects a structural effect as older patients have more chances to have had a long diagnosis journey because they have been alive for a longer period of time.

1. **Date of the confirmed diagnosis**

**Table 1** also shows that in our sample, the mean and median TDT increase when the diagnosis was confirmed more recently, including after 2010 and the introduction of Next Generation Sequencing (NGS). This can be the consequence of several structural effects which cannot be measured with the data available in our study:

- Respondents who have been diagnosed for a longer time (for instance before 2010) may be among those who survived their disease, for instance because their condition was less severe or because a diagnosis journey allowed them to access adapted care or treatments faster than others.
- Longer mean and median TDT after 2010 may show that new diagnostic technologies (including genetic technologies) allowed to end the diagnosis odyssey of patients who otherwise may have remained undiagnosed.

The authors of this paper have considered the possibility to reduce the dataset to respondents diagnosed after 2010 but think that this would only have hidden the structural effects of this variable. Graph A and Graph B provide visual representations that could help understand those structural effects:

- Graph A represents a theoretical distribution of some individual diagnosis journeys depending on the date of symptom onset and the date of confirmed diagnosis when all respondents are considered (n=6,507): in that case, mean TDT is 4.7 years.
- Graph B shows which types of diagnosis journeys would not have been considered if the authors had limited the dataset to respondents whose diagnosis was confirmed after 2010: the tendencies are similar, but mean and median TDT would be longer (respectively 5.2 years and 1.0 year) as respondents who were diagnosed before 2010 and had shorter mean and median TDT would no longer be considered. In addition, that option would not have allowed us to consider the determinants that contributed to those shorter and ‘older’ diagnosis journeys.

Hence, the date of the confirmed diagnosis was not included as a determinant of the TDT in this study: retrospective surveys are subject to too many structural effects for this variable. Longitudinal studies, or a repetition of retrospective surveys at different points in time, could provide more information on the evolution of TDT for PLWRD over time.

**Graph A. Theoretical repartition of individual diagnosis journey when considering all dates of confirmed diagnosis (n=6,507)**


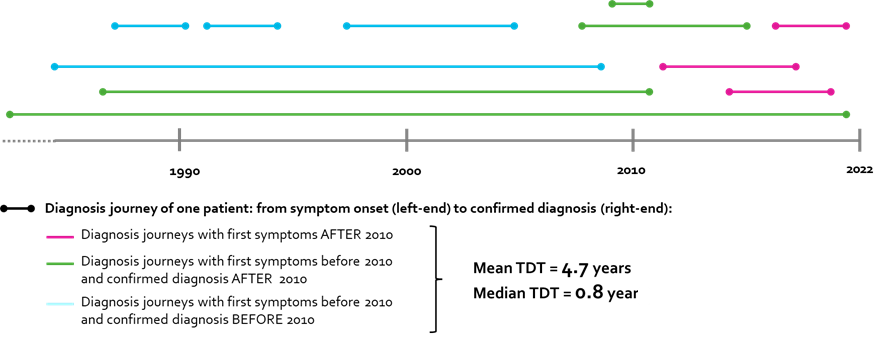


**Graph B. Theoretical repartition of individual diagnosis journey when considering only patients diagnosed after 2010 (n=4,451)**


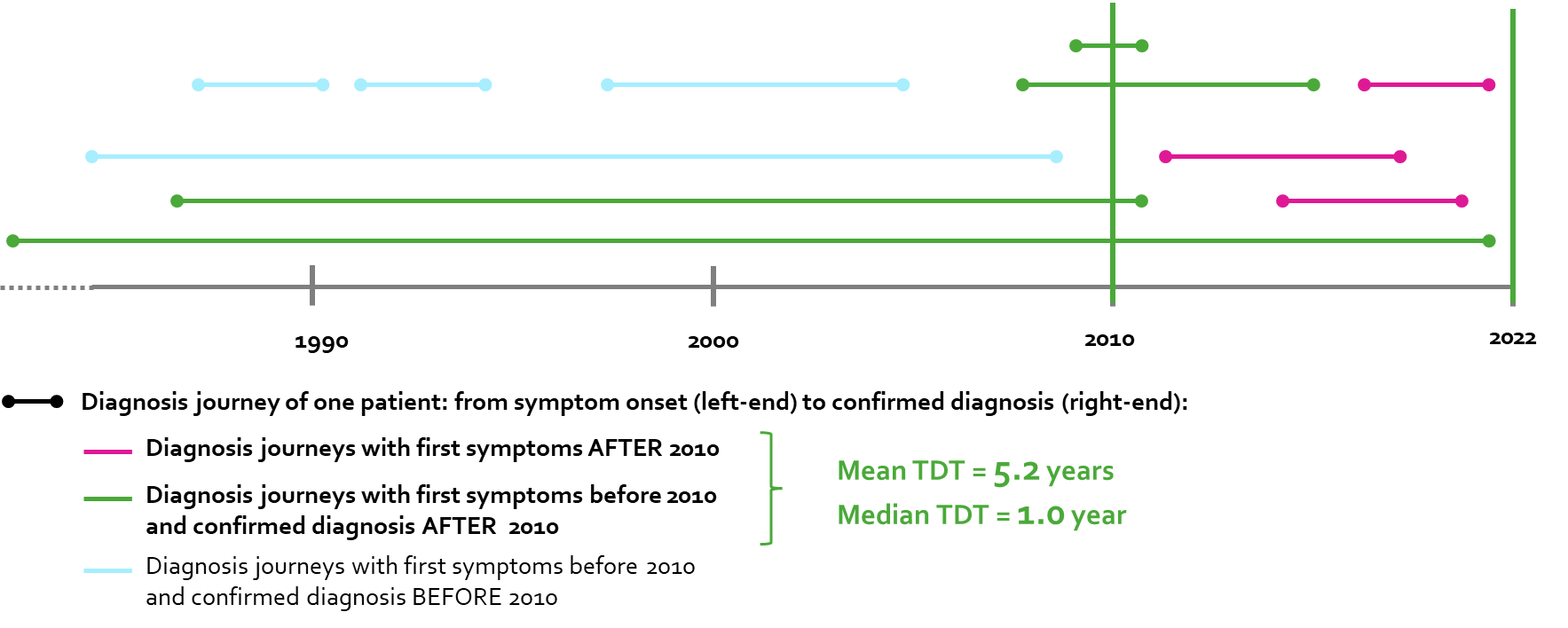


1. **Age of the patient at confirmed diagnosis**

The age of the patient at confirmed diagnosis was not included in this paper because:

- It is subject to the same structural effect as the variable ‘Age of the patient at the time of the study’: patients who were older when their diagnosis was confirmed have more chances to have had a long diagnosis journey because they have been alive for a longer period.
- It would have been a repetition of the information given by the age of the patient at symptom onset and of TDT (our variable of interest) as: *Age of the patient at confirmed diagnosis = Age of the patient at symptom onset + TDT.*

1. **Age of the patient at symptom onset**

The authors decided that the age of the patient at symptom onset would be the only time-related variable kept as a determinant of the TDT of PLWRD in this paper because it is at the beginning of the diagnosis journey, and it is subject to fewer structural effects than the time-related variables cited above when it is considered in a retrospective patient survey.
