# Supplementary material for: Time to diagnosis and determinants of diagnostic delays of people living with a rare disease: results of a Rare Barometer retrospective patient survey
Source: Eur J Hum Genet. 2024 May 16;32(9):1116–26. doi: 10.1038/s41431-024-01604-z (PMC11369105; doi:10.1038/s41431-024-01604-z)
Supplement: Supplementary file 6 — Additional File 6 [file 41431_2024_1604_MOESM6_ESM.docx]

**Additional file 6: Questionnaire (questions used in the paper)**

*Other questions from the questionnaire, and translation of all questions in the 27 languages of the survey, are available upon request to the corresponding author (*[*jessie.dubief@eurordis.org*](mailto:jessie.dubief@eurordis.org)*) or to* [*rare.barometer@eurordis.org*](mailto:rare.barometer@eurordis.org)*.*

1. **Rare Barometer framework**

Sociodemographic and recurring rare disease-related questions, and their translation in 23 languages, are available in the Rare Barometer questions repository accessible at this link: [*tiny.cc/speak_up_listen_up_guide*](http://tiny.cc/speak_up_listen_up_guide)

1. **Dates**

**As far as you remember, when…**

*Please indicate the month and year, for example 01/2000 for January 2000. If you do not remember the exact date, please indicate an approximate month and year.*

|  | Date | Not relevant | I don’t know |
| --- | --- | --- | --- |
| … did you or a healthcare professional first notice the symptoms of the rare disease or thought that something was wrong? | mm/yyyy |  |  |
| …did you first seek MEDICAL HELP regarding the symptoms of the rare disease? | mm/yyyy |  |  |
| …has the name of the rare disease, syndrome or malformation been CONFIRMED by appropriate genetic, clinical, medical imaging, molecular or biochemical tests (e.g biopsy, blood or urine test)? | mm/yyyy |  |  |

1. **Characteristics of the diagnosis journey (healthcare system)**

**How many different healthcare professionals did you consult (in person or virtually) while seeking a diagnosis?**

- 0
- 1
- between 2 and 4
- between 5 and 7
- between 8 and 10
- more than 10

**Did it ever happen that the symptoms of the rare disease were…**

|  | YES, one time | YES, several times | NO |
| --- | --- | --- | --- |
| …wrongly attributed to another physical disease? |  |  |  |
| …neglected, not taken seriously and/or considered as psychological? |  |  |  |

**As a consequence of the misdiagnosis, please tell us if you or the person you care for…**

|  | Definitely | Probably | Probably not | Definitely not | Don’t know / Not applicable |
| --- | --- | --- | --- | --- | --- |
| …could not access all the information needed to make informed reproductive choices such as planning whether or not to have children, or deciding whether or not to conduct prenatal tests |  |  |  |  |  |
| …received inappropriate care, treatment or surgery |  |  |  |  |  |
| …had a delayed access to the most appropriate care, treatment or surgery |  |  |  |  |  |
| …could not access appropriate care, treatment or surgery |  |  |  |  |  |
| …experienced worsening of the symptoms |  |  |  |  |  |

**Which tests were performed as part of the diagnosis of the rare disease?**

|  | Yes | No | Don’t know / don’t remember |
| --- | --- | --- | --- |
| Genetic test(s) looking for genetic changes (also called mutations or variants) |  |  |  |

**Have you ever needed a genetic test but could not access it because healthcare professionals were reluctant or not sufficiently informed?**

- Yes
- No
- Not applicable

**To your knowledge, the genetic test(s) that were conducted targeted…**

*Several answers possible if several tests*

- Only one gene
- Several genes at the same time (gene panel sequencing)
- The whole DNA (whole genome sequencing)
- All the genes (whole exome sequencing)
- A tumour (genetic profiling of a tumour)
- Other (epigenome, RNA, etc.)
- Don’t know

**I, or the person I care for, have been referred to a hospital unit specialised in the rare disease or group of rare diseases:**

- Yes
- No

1. **Characteristics of the diagnosis journey (family and support)**

**Family members were previously diagnosed with the same disease:**

- Yes
- No

**During your search for a diagnosis, were you proposed…**

|  | YES and enough to cover my needs | YES but it is/was not needed | YES but NOT enough to cover my needs | NO but it is/was NOT needed | NO but it is/was needed |
| --- | --- | --- | --- | --- | --- |
| …psychological support |  |  |  |  |  |
| …financial support including social security benefits |  |  |  |  |  |

1. **Characteristics of the rare disease and associated symptoms**

**Which parts of the body does the rare disease impact?**

***Please select all the answers corresponding to your situation***

# - lungs

# - kidneys

# - liver

# - heart

# - skin

# - bones

# - muscles, joints (musculoskeletal diseases)

# - eyes

# - craniofacial and ENT diseases

# - gastrointestinal tract (from the mouth to the anus)

# - vascular system

# - blood (haematological diseases)

# - reproductive and/or urinary systems

# - immune system (immunodeficiency, autoinflammatory and autoimmune diseases)

# - nervous system (brain, spine, nerves)

# - nerves and muscles (neuromuscular diseases)

# - hormonal functions (endocrine diseases)

# - metabolism

# - I don’t know

- Other, please specify

**Did the first symptoms include…**

|  | Yes | No | Don’t know |
| --- | --- | --- | --- |
| … behavioural disorders that cause problems in school, at home or in social situations |  |  |  |
| …clinical signs and symptoms that come and go |  |  |  |
| …sudden onset symptoms requiring urgent care |  |  |  |

**To your knowledge, what is the name of the rare disease?**

*Orphanet list of rare diseases in 7 languages and box for respondents to enter the name of their RD.*

1. **Post-diagnosis changes**

**Since receiving a diagnosis for the rare disease, how have the following aspects changed for you?** [To people living with a rare disease]

|  | …has improved | …has remained the same | …has gotten worse | I don’t know | Not applicable |
| --- | --- | --- | --- | --- | --- |
| Access to the most adapted care, treatments or surgery… |  |  |  |  |  |
| Understanding how the disease will progress… |  |  |  |  |  |
| Financial support including social security benefits… |  |  |  |  |  |
| Integration at school… |  |  |  |  |  |
| Integration at work… |  |  |  |  |  |
| Access to social services (e.g. social worker support, household chores support)… |  |  |  |  |  |
| Access to clinical trials… |  |  |  |  |  |
| Access to financial products, such as loans, mortgages, insurance… |  |  |  |  |  |
| Your social life… |  |  |  |  |  |

**Since receiving a diagnosis for the rare disease, how have the following aspects changed for the person you care for?** [To family members of people living with a rare disease]

|  | …has improved | …has remained the same | …has gotten worse | I don’t know | Not applicable |
| --- | --- | --- | --- | --- | --- |
| Access to the most adapted care, treatments or surgery… |  |  |  |  |  |
| Integration at school… |  |  |  |  |  |
| Access to clinical trials… |  |  |  |  |  |
| Access to financial products, such as loans, mortgages, insurance… |  |  |  |  |  |

**Since receiving a diagnosis for the rare disease, how have the following aspects changed for you?** [To family members of people living with a rare disease]

|  | …has improved | …has remained the same | …has gotten worse | I don’t know | Not applicable |
| --- | --- | --- | --- | --- | --- |
| Understanding how the disease will progress… |  |  |  |  |  |
| Financial support including social security benefits… |  |  |  |  |  |
| Integration at work… |  |  |  |  |  |
| Access to social services (e.g. social worker support, household chores support)… |  |  |  |  |  |
| Your social life… |  |  |  |  |  |
